# Cerebral oscillatory activity during simulated driving using MEG

**DOI:** 10.3389/fnhum.2014.00975

**Published:** 2014-12-16

**Authors:** Kotoe Sakihara, Masayuki Hirata, Kazutoshi Ebe, Kenji Kimura, Seong Yi Ryu, Yoshiyuki Kono, Nozomi Muto, Masako Yoshioka, Toshiki Yoshimine, Shiro Yorifuji

**Affiliations:** ^1^Department of Functional Diagnostic Science, Graduate School of Medicine, Osaka UniversitySuita, Japan; ^2^Department of Clinical Laboratory Science, Faculty of Medical Technology, Teikyo UniversityItabashi-ku, Japan; ^3^Department of Neurosurgery, Graduate School of Medicine, Osaka UniversitySuita, Japan; ^4^Frontier Research Center, Toyota Central R&D Labs., Inc.,Nagakute, Japan; ^5^Human System Integration Group, Vehicle Engineering Development Division, Toyota Motor CorporationToyota, Japan

**Keywords:** oscillation, car driving, magnetoencephalography

## Abstract

We aimed to examine cerebral oscillatory differences associated with psychological processes during simulated car driving. We recorded neuromagnetic signals in 14 healthy volunteers using magnetoencephalography (MEG) during simulated driving. MEG data were analyzed using synthetic aperture magnetometry to detect the spatial distribution of cerebral oscillations. Group effects between subjects were analyzed statistically using a non-parametric permutation test. Oscillatory differences were calculated by comparison between “passive viewing” and “active driving.” “Passive viewing” was the baseline, and oscillatory differences during “active driving” showed an increase or decrease in comparison with a baseline. Power increase in the theta band was detected in the superior frontal gyrus (SFG) during active driving. Power decreases in the alpha, beta, and low gamma bands were detected in the right inferior parietal lobe (IPL), left postcentral gyrus (PoCG), middle temporal gyrus (MTG), and posterior cingulate gyrus (PCiG) during active driving. Power increase in the theta band in the SFG may play a role in attention. Power decrease in the right IPL may reflect selectively divided attention and visuospatial processing, whereas that in the left PoCG reflects sensorimotor activation related to driving manipulation. Power decreases in the MTG and PCiG may be associated with object recognition.

## INTRODUCTION

Operating a motor vehicle is a human superior ability that involves many psychological processes of concentration, attention, motor control, and visuomotor integration. We aimed to detect the oscillatory activities associated with these psychological processes during simulated driving. In an electroencephalography (EEG) study using driving simulators, Laukka et al. (1995) showed that theta band increase in the medial prefrontal cortex (MPC) reflects concentration, and Schier (2000) showed that alpha band decrease in the posterior area reflects attention. These signal power increases and decreases are called oscillatory differences, and they are responses to specific events (Pfurtscheller and Lopes da Silva, 1999a). Oscillatory decreases and increases in a specific frequency band are associated with neural activity (Pfurtscheller and Lopes da Silva, 1999b). Oscillatory differences have been used for evaluating neuronal activity during cognitive tasks (Pfurtscheller and Lopes da Silva, 1999a) associated with attention and language (Ishii et al., 1999; Hirata et al., 2007; Maratos et al., 2007). Previous EEG studies with a driving simulator were conducted with relatively low spatial resolution (at the hemisphere or lobar level). To detect oscillatory differences with high resolution, we used magnetoencephalography (MEG) whose resolution is equal to that of fMRI (Hirata et al., 2007). In addition, previous neuroimaging studies with driving simulators lacked driving reality. They used a simple joystick or a game pad controller (Walter et al., 2001; Uchiyama et al., 2003) for driving. In contrast, in the present study, we used a steering wheel, an accelerator, and a brake to provide a realistic driving experience. We hypothesized that oscillatory differences in frontal and parietal regions associated with concentration and attention required for driving could be detected.

## MATERIALS AND METHODS

### SUBJECTS

Fourteen healthy volunteers (2 men and 12 women; age range, 21–57 years) participated in this study. They were all right-handed, as confirmed using the Edinburgh Handedness Inventory (Oldfield, 1971). The subjects were medically evaluated prior to the study, and all were found to be in good physical condition and free from any pathological sleep disorder. None of the subjects had a history of neurological or psychiatric disease, and all had normal or corrected-to-normal vision. Some subjects did not have a license to drive, but practiced sufficiently prior to the study to get accustomed to simulated driving. The subjects were instructed to have sufficient sleep before the experiment so that they were fully awake and not fatigued at the beginning of the experiment. Informed consent was obtained from all subjects prior to study initiation. All experimental procedures were in accordance with the guidelines of the Declaration of Helsinki.

### EXPERIMENTAL PROTOCOL

**FIGURE 1:**
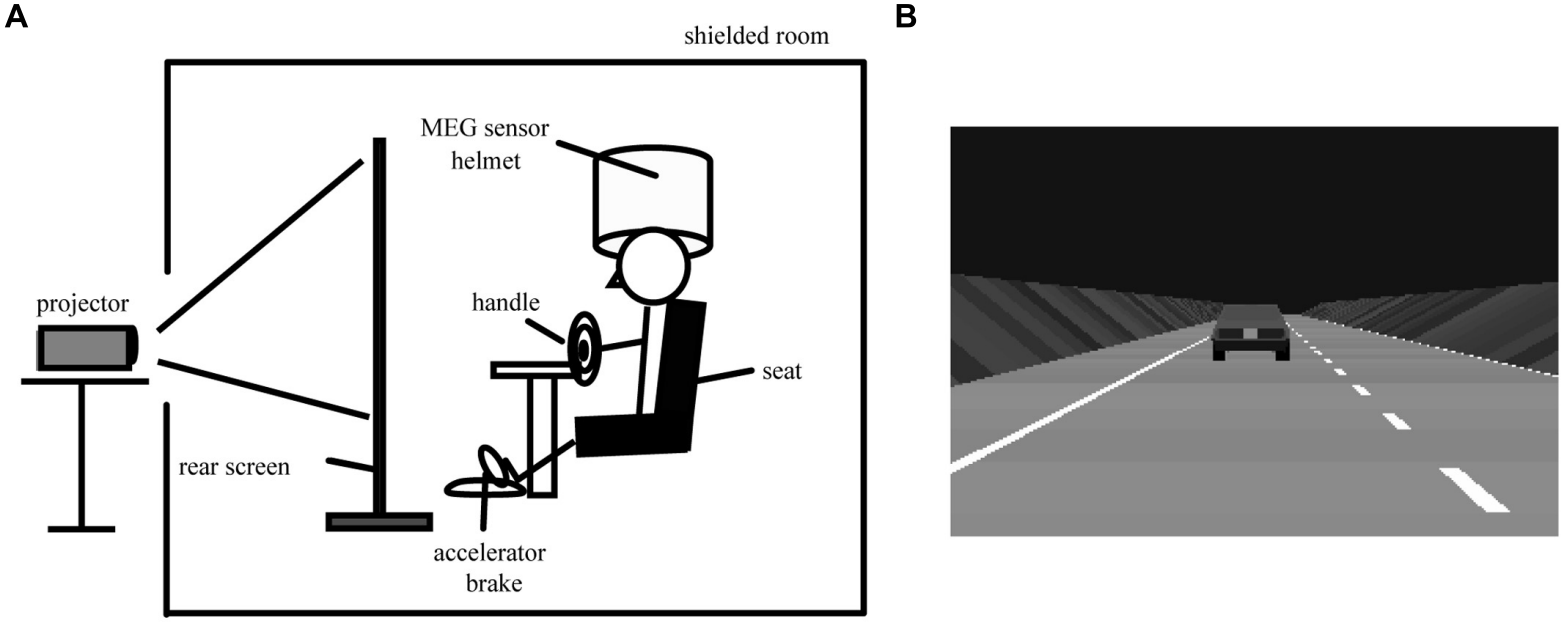
**Experimental overview and simulated driving animation. (A)** Experimental overview during simulated driving. **(B)** A simulated driving animation projected onto the rear projection screen. The driving animation is actually colored. During passive viewing, the subjects were asked to view the car ahead, which was moving at a constant speed of 80 km/h. During active driving, the subjects were asked to use a steering wheel, an accelerator, and a brake to maintain a constant distance behind the car ahead and to stay in their lane.

The driving simulation system was set up in a magnetically shielded room. A driving simulator equipped with a steering wheel, an accelerator, and a brake (SideWinder Racing Wheel, Microsoft Co., Washington, DC, USA) was remodeled to be compatible with MEG recording (**Figure 1A**). Ferromagnetic parts were replaced with non-magnetic materials. The subjects were seated on a comfortable chair with their hands on the steering wheel (**Figure 1A**) and were asked to perform simulated driving tasks. A pneumatic cuff was placed between the subjects’ heads and the MEG head gantry to prevent movement of the subjects’ heads. A large rear projection screen (80-inch) was placed 1.3 m in front of the subjects, and a simulated driving animation was projected onto the screen from a liquid crystal projector placed outside the shielded room. The shielded room was dark, the driving animation simulated a freeway at night (**Figure 1B**), and the driving course had two lanes that were mostly straight with gentle curves, no intersections, no signals, and no pedestrians. Oncoming cars appeared occasionally.

### SIMULATED DRIVING

**FIGURE 2:**
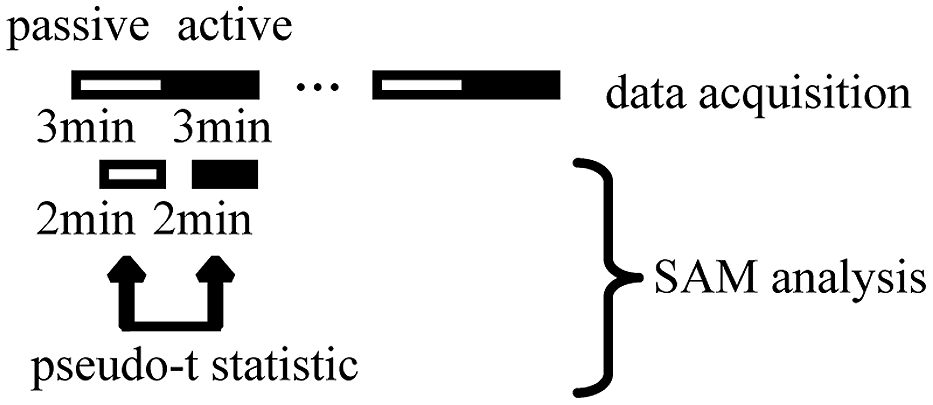
**Overall scheme of simulated driving, magnetoencephalography data acquisition, and synthetic aperture magnetometry (SAM) analysis.** Each cycle of passive viewing and active driving lasted 3 min. The whole task comprised cycles of passive viewing and active driving that were alternately repeated 10 times. The total experimental duration was 60 min. Within a 3-min driving period, the first minute was used for measuring head position. We excluded this minute from the analysis to minimize the effect of a change in wakefulness due to task change. The remaining 2 min were used for SAM analysis. We calculated pseudo *t* statistics during the statistical analysis of grouped SAM data.

The simulated driving task consisted of an active car-following session (active session) and a passive car-following session (passive session) for 1 h. The task was set up in a block design, the same as that for fMRI (Uchiyama et al., 2003). Three minutes of passive session was followed by 3 min of active session, with this cycle being repeated 10 times without intermission (**Figure 2**). In the passive session, the car ahead moved at a constant speed. The subjects viewed a driving simulation in which the subject’s car automatically followed the car ahead at a constant distance with constant speed. In the active session, the subjects manipulated the accelerator and brake to follow the car ahead while maintaining a constant distance between the cars and maintaining their position in the lane. Our subjects practiced sufficiently with the driving simulator to become accustomed to the driving task prior to the experiment. We expected that general adaptation to the driving simulator and the skill of the subject would reach a plateau and that the effects of adaptation and driving skill on the results would be minimal. The same task order was maintained for each subject. We expected that the effect of the order of passive and active sessions would be minimized by consecutive repetition without a pause, so that the order of the driving tasks did not distract the subject’s attention during the experiment.

### MEG RECORDINGS

Neuromagnetic signals were recorded using a 64-channel whole-head MEG system equipped with SQUID gradiometers (NeuroSQUID Model 100, CTF Systems Inc., Port Coquitlam, BC, Canada). Localization of each subject’s head relative to the sensor array was measured with three coils affixed to the nasion and bilateral preauricular points at the beginning of every active driving and passive viewing trial. The magnetic field signals were digitally recorded at a sampling rate of 625 Hz with an online band pass filter from 1 to 200 Hz. Notch filters at 60, 120, and 180 Hz were used to eliminate AC line noise. Ten sets of passive viewing and active driving data were collected for each subject. During the 3 min of driving time in each cycle, the first minute was used for measuring the subject’s head position and the remaining 2 min were used for MEG data recording and synthetic aperture magnetometry (SAM) analysis. Head position coordinates were recorded every 5 min, and absence of head movement artifacts was confirmed. Given that our task simulated driving on a highway, limb movements to manipulate a wheel and a brake were minimal, and we confirmed that EMG artifacts resulting from body movements were excluded from MEG data. We excluded ocular artifacts using SAM analysis, as signals from unwanted sources were suppressed.

### MRI ACQUISITION

For each subject, MRI scans were obtained using either two 1.5-T MR imaging systems (Signa EXCITE HD 1.5T, GE Medical Systems, Milwaukee, WI; MAGNETOM Vision plus, Siemens, Berlin, Germany) or one 3-T MR imaging system (Signa EXCITE HD 3T, GE Medical Systems, Milwaukee, WI, USA). Individual MRI data comprised T1-weighted sequences in 130 sagittal slices (1.4-mm thickness), with fiducial skin markers placed at the nasion and bilateral preauricular points. Three markers (high-contrast oil capsules) were attached to the same positions as the coils during MEG recording. The MEG data were superimposed onto individual MRI images with an anatomical accuracy of a few millimeters because of registration of the head position at these three points.

### SAM ANALYSIS

To evaluate local oscillatory differences associated with psychological processes during driving, it is necessary to detect multiple activated areas in a wide frequency band. To improve spatial resolution, we used SAM analysis (Robinson and Vrba, 1998; Taniguchi et al., 2000). In SAM analysis, a linearly constrained minimum-variance beamformer calculates the source power by forming a linear combination of sensors that suppress signals from unwanted sources (environmental and brain noise) without attenuation of signal from the target region. SAM analysis is ideally suited for analysis of event-related differences in cortical rhythms in a specific frequency band without averaging and can be used to depict the spatiotemporal distributions of oscillatory differences as statistical parametric maps with high spatial resolution comparable to that of fMRI (Hirata et al., 2007). Oscillatory differences can be estimated statistically by voxel-to-voxel comparisons in the specified frequency bands. Moreover, it is possible to statistically calculate group effects (Singh et al., 2003; Furlong et al., 2004; Gaetz and Cheyne, 2006; Maratos et al., 2007) to exclude differences among individuals. A multisphere head model was used. The region of interest was set to include the entire cerebral cortex with a 5-mm voxel resolution, and the current density of each voxel was estimated by SAM. Based on our hypotheses described in the Introduction section, we specifically analyzed brain areas that play important roles in concentration, attention, motor control, and visuomotor integration. MEG signals were divided into four frequency bands: theta (4–8 Hz), alpha (8–13 Hz), beta (13–25 Hz), and low gamma (25–50 Hz). In our simulated driving task, the subjects were assumed not to move through sleep stage 3, given that the simulated driving task cannot be performed at sleep stage 3; thus, we dropped the delta band (1–4 Hz) for frequency analysis. Oscillatory differences in the current density in each voxel during the active session (1–3 min) and passive session (1–3 min) were assessed using a pseudo *t* statistic, enabling a three-dimensional SAM image of cortical activity to be generated for each individual. Power increases and decreases are highly variable between subjects. As a result, inferences based on the classical *t-*test may not be optimal because the variance denominator needed for the *t* statistic is poorly estimated. In context imaging, this situation results in high spatial noise on the variance map. To reduce this noise, we spatially smoothed the variance map (but not the activation map) before using it for calculating voxel-wise *t* statistics (Nichols and Holmes, 2001). Furthermore, there is some evidence that the magnitude of power increases and decreases is not normally distributed across subjects (Burgess and Gruzelier, 1999). Pseudo *t*-statistic images formed with smoothed variance estimators are usually smooth. The noise from the variance image was smoothed, whereas the signal was not. We accordingly used an analysis that allowed statistical inference and correction for multiple comparisons using estimators such as the smoothed variance pseudo *t* statistic, without an assumption of normality. We adapted statistical group analysis for the SAM data. First, the functional volumes computed by SAM were aligned with the individual anatomical MR images using SPM2^1^. These functional volumes were spatially normalized into a standard Montreal Neurological Institute (MNI; T1) template space using SPM2 implemented in MATLAB^2^, after which they were resampled to produce images with 2 mm × 2 mm × 2 mm resolution. Next non-parametric permutation analyses were performed with the SnPM toolbox^3^ to assess significant group effects for voxel-level inference using a multiple-subject single-condition design. Probability maps for significant effects (*p* < 0.05, corrected), referred to as grouped SAM images, were visualized using mri3dX^4^. Given that SPM2 uses standard brains from the MNI, the MNI coordinates were converted to Talairach coordinates using nonlinear transformation (Lancaster et al., 2000) in the mri3dX software package. The neuroanatomical locations of significant effects were determined using a Talairach database (Lancaster et al., 2000).

### SPECTRAL ANALYSIS OF MEG DATA

The power spectrum of MEG data was analyzed during passive and active sessions by fast Fourier transform using brain electrical source analysis (BESA) software (Version 5.1, MEGIS Software GmbH, Gräfelfing, Germany). The time window for the analysis was 2 min and the analyzed frequency bands were the theta (4–8 Hz), alpha (8–13 Hz), beta (13–25 Hz), and low gamma (25–50 Hz) bands. The related-sample Friedman’s two-way analysis of variance (ANOVA) by ranks with *post hoc* multiple comparison was performed to estimate the temporal differences of power spectra. SPSS software (15.0J, IBM, Armonk, NY, USA) was used for statistical analyses.

### RATING OF DROWSINESS AND FATIGUE

The experiment time was rather long in comparison with that used in previous driving studies (Bowyer et al., 2009; Hsieh et al., 2009); thus, prior to the experiments without MEG recording, we tested the subjects’ drowsiness and fatigue by objective and subjective ratings. To evaluate objective drowsiness, we recorded each subject’s facial expressions using a video camera during simulated driving and estimated the drowsiness level on the basis of the New Energy and Industrial Technology Development Organization (NEDO) standard (NEDO, 1999): D0 was defined as awake, D1 as slightly drowsy, D2 as drowsy, D3 as very drowsy, and D4 as extremely drowsy. This Japanese standard is most frequently used in Japan to evaluate drowsiness during driving and corresponds well with the American standard (Wierwille and Ellsworth, 1994). The drowsiness level based on facial expression was determined by two different experimenters. These drowsiness levels were recorded every 4 min. The Friedman test was performed to assess the temporal change and was followed by the Wilcoxon matched-pairs test. When a subject left the lane because of drowsiness, the recording was stopped. Half of the subjects dozed off and drifted off course by 39 min, and the analysis time was accordingly set at 39 min.

In the subjective rating, the subjects rated drowsiness and fatigue on a visual analog scale from 0 to 10. The drowsiness scale ranged from 0 (indicating being awake) to 10 (indicating falling asleep). The fatigue scale ranged from 0 (indicating being alert) to 10 (indicating being exhausted). The rating time point was 30 and 60 min since driving task stared. The rating scores of drowsiness and fatigue ratings were statistically compared using the Wilcoxon matched-pair signed-rank test.

### CORRELATION BETWEEN MEG DATA AND SUBJECTIVE DROWSINESS AND FATIGUE

The correlation between MEG power value and subjective drowsiness and fatigue was estimated. The magnetic field signal intensity was evaluated for each subject during active driving using BESA as described above. Pearson’s product–moment correlation analysis was used to calculate the correlation.

## RESULTS

### OSCILLATORY DIFFERENCES DURING ACTIVE DRIVING

**FIGURE 3:**
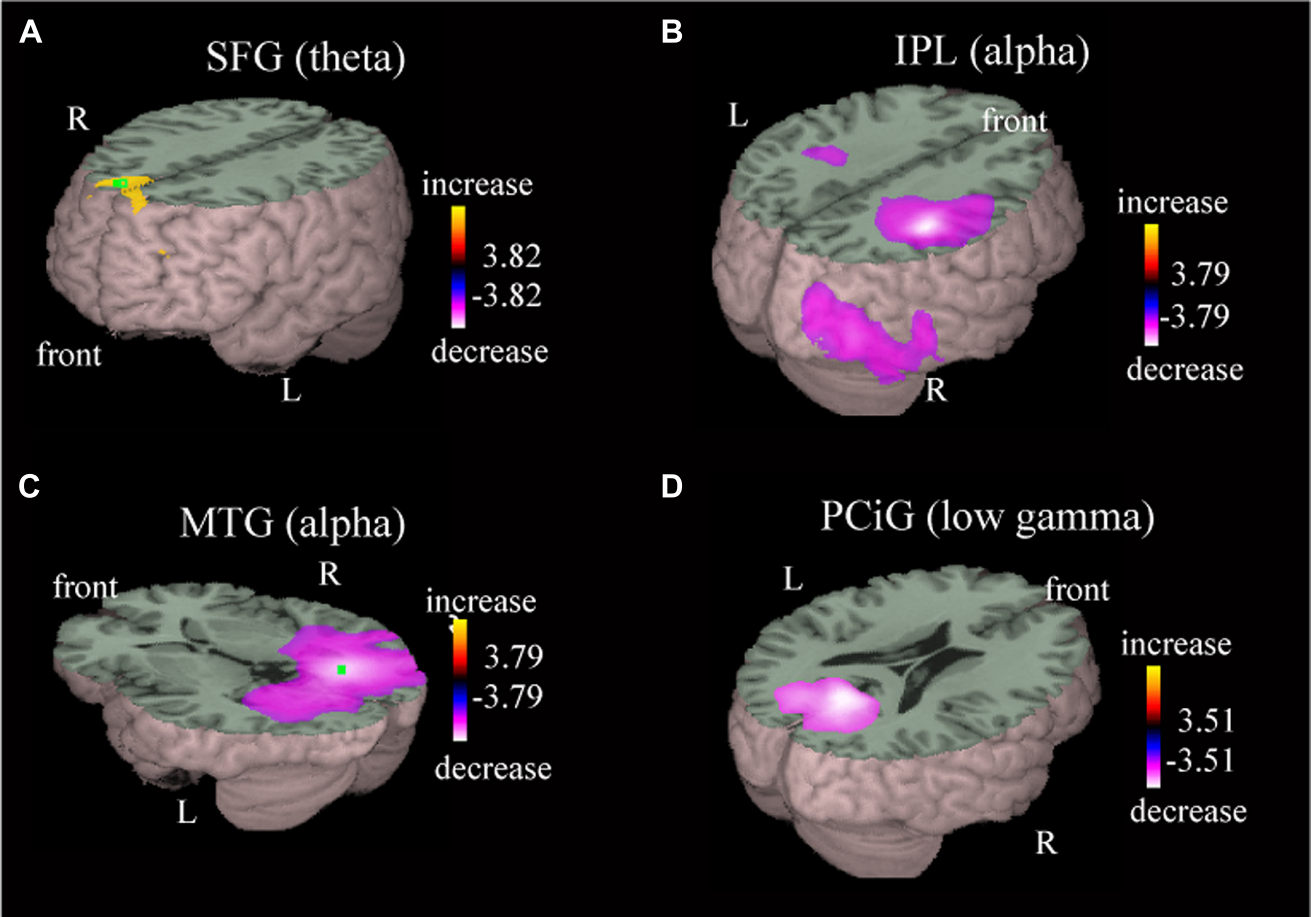
**Representative images of group data showing power increase and power decrease.** The presented areas and the frequency bands are the superior frontal gyrus in the theta band **(A)**, inferior parietal lobe in the alpha band **(B)**, middle temporal gyrus in the alpha band **(C)**, and posterior cingulate gyrus in the low gamma band **(D)**. Group effects are shown as orange-to-yellow colors or blue-to-white colors overlaid on three-dimensional surface rendering normalized onto a template brain. The respective pseudo *t* value scale is congruent with a *p* value of 0.05 (family-wise error corrected).

Local oscillatory differences were detected during active driving in comparison with passive viewing in multiple brain regions, including the MPC, postcentral gyrus (PoCG), posterior parietal area, temporo-occipital area, and occipital gyrus (**Table 1**). Power increase in the theta band was observed in the superior frontal gyrus (SFG) during active driving in comparison with passive viewing [**Figure 3A**; *p* < 0.05, family-wise error (FWE) corrected]; theta power increase was also observed in the medial frontal gyrus (MFG; **Table 1**). Power decreases in the alpha and beta bands were observed in the right inferior parietal lobe (IPL; *p* < 0.05, FWE corrected; **Figure 3B**); the *t* value was more prominent in the right hemisphere than in the left hemisphere. We also observed power decrease in the precuneus (*p* < 0.05, FWE corrected). Alpha power decrease in the middle temporal gyrus (MTG) and low gamma power decrease in the posterior cingulate gyrus (PCiG) were observed (*p* < 0.05, FWE corrected; **Figures 3C,D**). Gamma power decrease was also observed in the superior occipital gyrus. In addition, alpha power decrease was detected in the PoCG.

**Table 1:** *T* values of oscillatory differences in the main frequency bands in each region.

			Coordinates (mm)			Voxel level	Cluster size
Cerebral region (BA)^#^	Frequency band^&^	Oscillatory difference	*x*	*y*	*z*	*t* value*	*K*
lt SFG (6)	Theta	Power increase	–22	32	57	4.77	492
rt SFG (9)	Theta	Power increase	16	46	35	4.28	1071
rt MFG (9)	Theta	Power increase	2	55	41	4.23	1071
lt PoCG (3)	Alpha	Power decrease	–46	–15	54	4.79	1408
lt PoCG (1)	Beta	Power decrease	–44	–30	60	4.12	104
rt IPL (40)	Alpha	Power decrease	44	–31	35	6.49	37,075
rt IPL (40)	Beta	Power decrease	42	–32	29	6.14	40,304
lt IPL (40)	Gamma	Power decrease	–53	–54	49	3.63	215
lt PCU (19)	Gamma	Power decrease	–28	–80	41	3.68	215
rt MTG (19)	Alpha	Power decrease	22	–58	–3	6.11	37,075
rt SOG (19)	Gamma	Power decrease	46	–78	30	3.75	37
lt PCiG (23)	Beta	Power decrease	–4	–58	14	6.16	40,304
lt PCiG (23)	Gamma	Power decrease	–2	–57	16	4.62	5774

*^∗^All *t* values are FWE corrected. PFWE value < 0.05*.

*^#^BA, Brodmann area; SFG, superior frontal gyrus; MFG, medial frontal gyrus; IPL, inferior parietal lobe; PCU, precuneus; MTG, middle temporal gyrus; SOG, superior occipital gyrus; PCiG, posterior cingulate gyrus; PoCG, postcentral gyrus; lt, left; rt, right*.

*^&^Frequency bands: theta (4–8 Hz), alpha (8–13 Hz), beta (13–25 Hz), and low gamma (25–50 Hz)*.

### DIRECT COMPARISON OF OSCILLATORY DIFFERENCES DURING ACTIVE DRIVING IN TEMPORAL VARIATIONS

**FIGURE 4:**
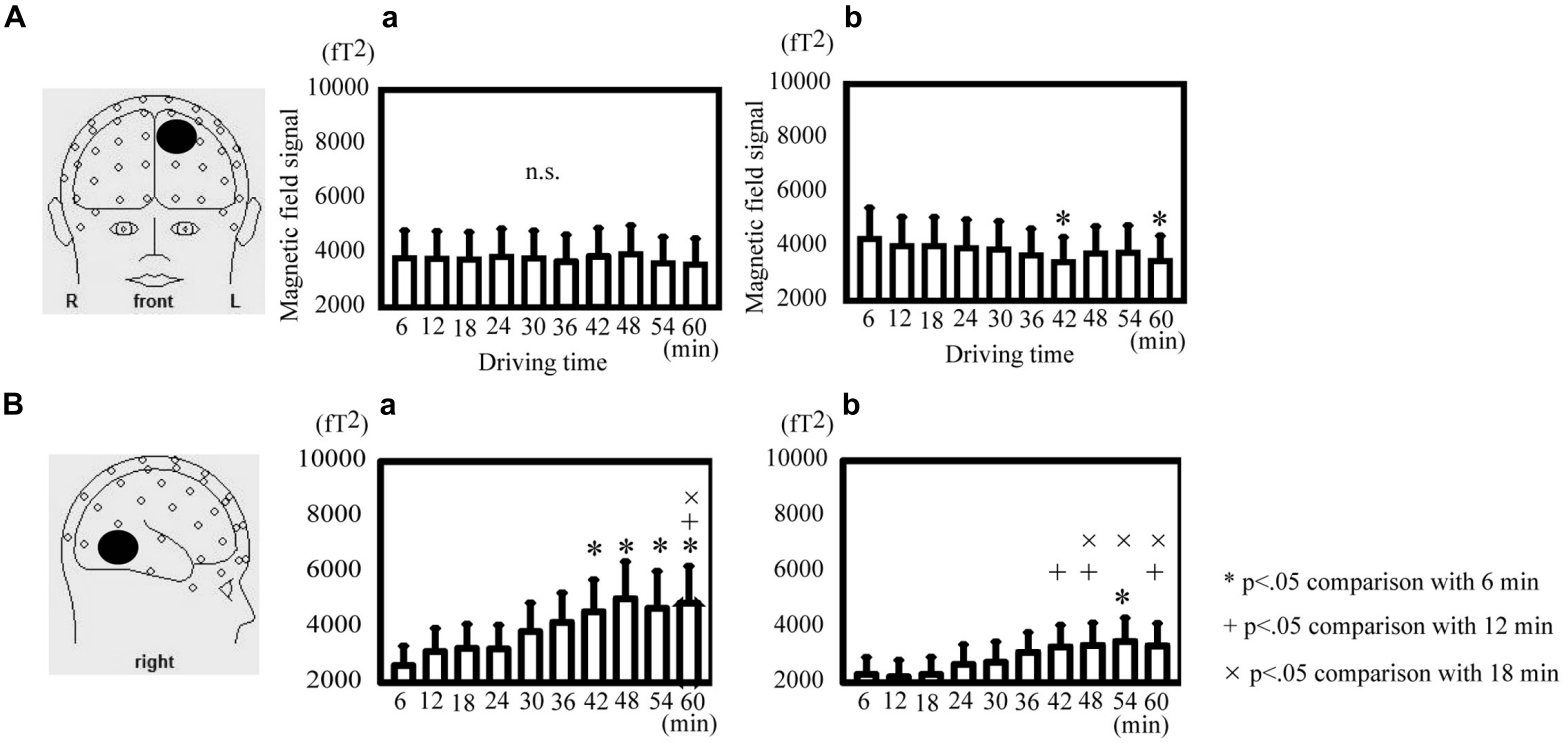
**Direct comparison of oscillatory differences during active driving in temporal variations. (A)** Oscillatory activity analyzed in the theta band in the superior frontal gyrus during passive viewing (a) and active (b) driving. **(B)** Oscillatory activity analyzed in the alpha band in the middle temporal gyrus during passive viewing (a) and active (b) driving. Error bars indicate standard errors. *p < 0.05 comparison with the time of 6 min, ^+^*p* < 0.05 comparison with the time of 12 min,^×^*p* < 0.05 comparison with the time of 18 min (Friedman analysis of variance).

The theta power in the SFG decreased significantly at 42 and 60 min in comparison with that at 6 min during active driving [Friedman ANOVA, χ^2^(9, *N* = 14) = 35.83, *p* < 0.05; **Figure 4A–b**] but not during passive viewing (**Figure 4A–a**). The alpha power in the MTG significantly increased at 54 min in comparison with that at 6 min; at 42, 48, and 60 min in comparison with that at 12 min; and at 48, 54, and 60 min in comparison with that at 18 min during active driving [Friedman ANOVA, χ^2^(9, *N* = 14) = 50.45, *p* < 0.05; **Figure 4B–b**]. During passive viewing, the alpha power in the MTG significantly increased at 42, 48, 54, and 60 min in comparison with that at 6 min and at 60 min in comparison with that at 12 and 18 min [Friedman ANOVA, χ^2^(9, *N* = 14) = 42.13, *p* < 0.05; **Figure 4B–a**].

### OBJECTIVE AND SUBJECTIVE DROWSINESS AND FATIGUE

**FIGURE 5:**
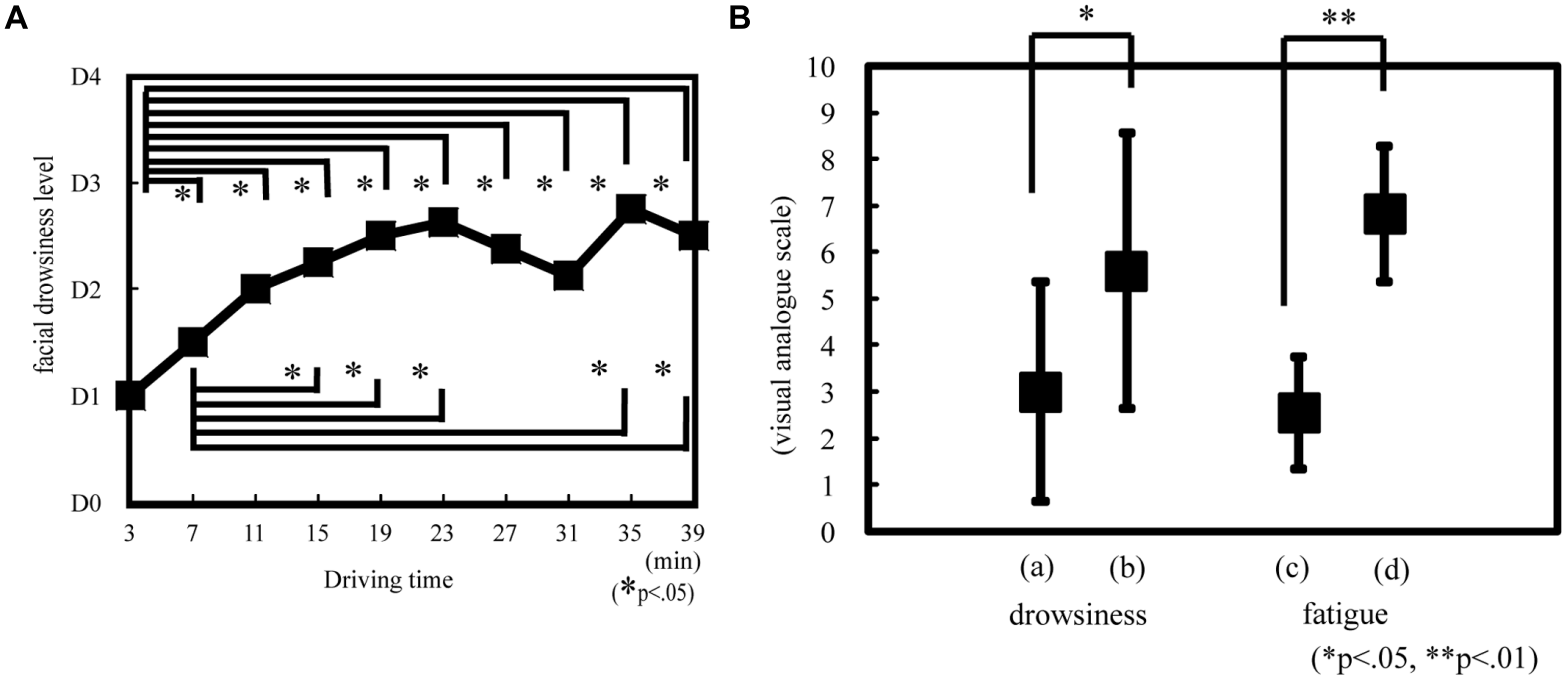
**Drowsiness or fatigue level according to objective and subjective evaluation.** Objective drowsiness **(A)** was evaluated on the basis of facial expression as estimated according to the New Energy and Industrial Technology Development Organization (NEDO) standards during driving. D0, awake; D1, slightly drowsy; D2, drowsy; D3, very drowsy; and D4, extremely drowsy. *p < 0.05. Subjective drowsiness **(B)** and fatigue was evaluated on the basis of visual analog scale rating. The subjects reported drowsiness and fatigue rating after 30 and 60 min. On the drowsiness scale, 0 indicated that the subject was awake and 10 indicated that the subject was sleepy. On the fatigue scale, 0 indicated that the subject was alert and 10 indicated that the subject was exhausted. (a) Drowsiness at 30 min. (b) Drowsiness at 60 min. (c) Fatigue at 30 min. (d) Fatigue at 60 min. *p < 0.05, **p < 0.01.

The objective drowsiness level showed a temporal significant increase during the experiment [Friedman ANOVA, χ^2^(9, *N* = 14) = 29.50, *p* < 0.05; **Figure 5A**]. Drowsiness significantly increased at 7, 11, 15, 19, 23, 27, 31, 35, and 39 min compared with that at 3 min (Wilcoxon matched-pair signed-rank test, *p* < 0.05); it also significantly increased at 15, 19, 23, 35, and 39 min compared with that at 7 min (Wilcoxon matched-pair signed-rank test, *p* < 0.05; **Figure 5A**). The objective drowsiness level increased and reached a plateau at 30 min. The subjective drowsiness and fatigue level was significantly greater at 60 min than at 30 min (Wilcoxon matched-pair signed-rank test: drowsiness, *p* < 0.05, *r* = 0.56; fatigue, *p* < 0.01, *r* = 0.89; **Figure 5B**).

### CORRELATION BETWEEN SUBJECTS’ DROWSINESS AND FATIGUE AND OSCILLATORY DIFFERENCES

**FIGURE 6:**
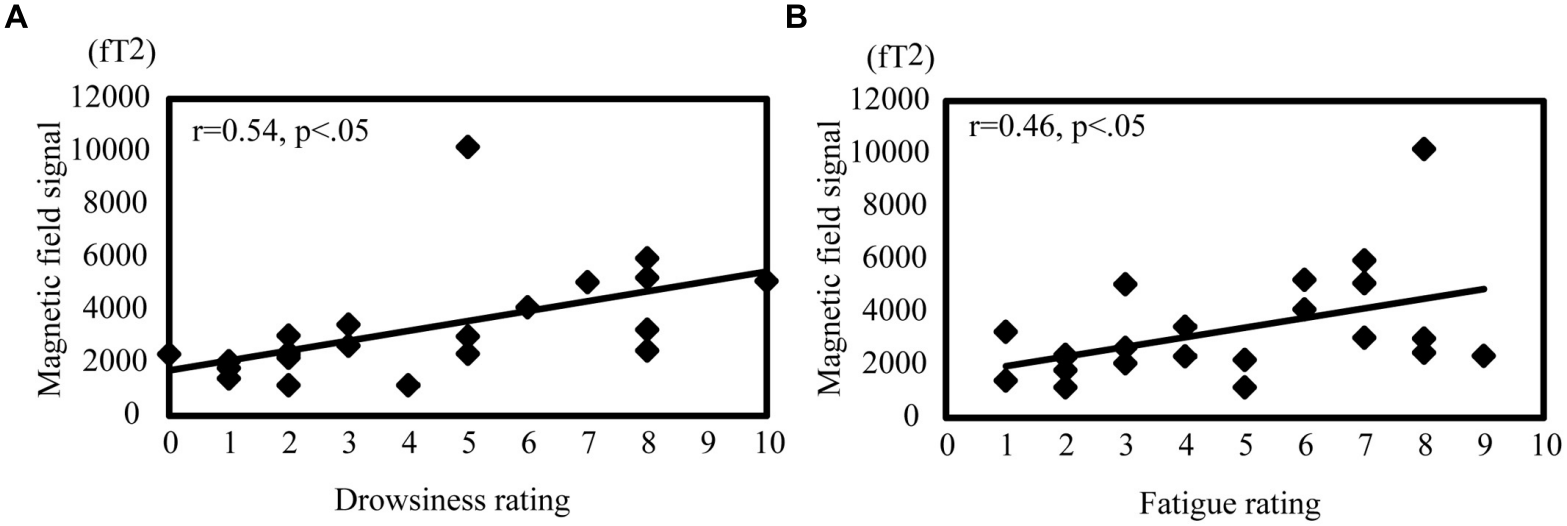
**Correlation between oscillation power and drowsiness and fatigue ratings during passive viewing.** Correlation between alpha band activity in the MTG and drowsiness **(A)** and fatigue **(B)**. The correlation coefficient was noted as r. |r| > 0.4 represents a moderate correlation and |r| > 0.7 represents a strong correlation.

Pearson’s product–moment correlations were calculated to evaluate the relationship between each subject’s rating of drowsiness and fatigue and the magnetic signal intensity during passive viewing and active driving (**Figure 6**). In passive viewing, the alpha value in the MTG showed a moderate positive correlation with drowsiness (*r* = 0.54, *p* < 0.05; **Figure 6A**) and fatigue (*r* = 0.46, *p* < 0.05; **Figure 6B**). Other brain regions showed no significant correlation with drowsiness and fatigue in passive viewing or active driving.

## DISCUSSION

We detected oscillatory differences in the SFG, IPL, PoCG, MTG, and PCiG during simulated driving, and these differences were interpreted as correlates of an activated cortical area associated with cognitive function. These brain regions were consistent with those reported in prior EEG and fMRI studies of simulated driving. To our knowledge, we are the first to demonstrate oscillatory differences in cognitive function using a driving simulator and MEG.

### THE ATTENTION NETWORK

#### Anterior attention network

In the present study, theta power increase was identified in the SFG and MFG, which are regions of the MPC. Theta band activity was induced in the MPC during simulated driving (Laukka et al., 1995). The induction of theta band oscillation in the MPC, known as frontal midline theta rhythm (Fm theta), has been proposed to occur during various mental tasks, including mental arithmetic and tracing a maze (Ishihara and Yoshi, 1972; Iramina et al., 1996; Sasaki et al., 1996; Gevins et al., 1997; Smith et al., 1999; Stam, 2000). Fm theta was different from the theta enhancement at sleep stage 3. As sleep stage 3 is a deep sleep condition, people do not respond to external stimuli. In the present study, as the subjects continued to drive, they never moved through sleep stage 3. Fm theta has been suggested to reflect attention levels (Kubota et al., 2001; de Araujo et al., 2002; Jensen and Tesche, 2002; Mizuhara et al., 2004; Otmani et al., 2005; Missonnier et al., 2006; Deiber et al., 2007; Mitchell et al., 2008). In the present study, the theta power increase observed in the MPC was most likely Fm theta and reflected the attention response induced by driving. Fm theta decreases with attention levels (Ishii et al., 1999; Kahana et al., 1999; Klimesch, 1999; Krause et al., 2000; Aftanas and Golocheikine, 2001). MFG is positively correlated with arousal levels (Field, 2005). When arousal level increases as a consequence of the activation of working memory, theta power increases throughout the frontal cortex (Cohen, 1992). Two attention networks that rely on interactions with the arousal system have been proposed on the basis of previous research (Posner et al., 1984): the anterior and posterior attention networks. The anterior attention network apparently involves detection of sensory targets and is strongly reliant on the anterior cingulate cortex (Cohen, 1992).

#### Posterior attention network

A prominent alpha power decrease was observed in the right IPL in our study. An oscillatory power decrease is considered to represent cortical activation (Pfurtscheller, 2001; Ihara et al., 2003). Recent fMRI studies using simulated driving suggest that the IPL is activated when subjects try to keep a safe distance from a car ahead of them (Uchiyama et al., 2003) or when they try to turn a corner or avoid a collision (Spiers and Maguire, 2007). IPL activation during driving is proposed to reflect selectively divided attention to visual motion (Spiers and Maguire, 2007). An alpha band power decrease in the right IPL has been reported during simulated driving (Schier, 2000), and the authors proposed that this power decrease reflected attention activity. Alpha activity in the posterior region is known as the posterior attention network (Posner et al., 1984).

### VISUOSPATIAL INFORMATION AND SENSORIMOTOR CONTROL

The right parietal region is also important for visuospatial processing and plays a role in the dorsal pathway for visual processing of “where” information (Mishkin et al., 1983; Sergent et al., 1992; Ungerleider and Haxby, 1994). A lesion in the right parietal region causes topographical disorientation (Aguirre and D’Esposito, 1999). Further, an alpha band in the left PoCG reflects activity in the sensorimotor area (Pfurtscheller and Lopes da Silva, 1999a). Oscillatory differences in the right IPL and PoCG may reflect visuospatial and sensorimotor processes.

### HIGHER-ORDER VISUAL PROCESSING

The MTG and PCiG are located in the occipitotemporal area, which is believed to play an important role as a gateway from lower to higher visual processes (Puce et al., 1996; Tarkiainen et al., 2002; Jobard et al., 2003), in particular the identification of landmarks and places (Aguirre and D’Esposito, 1997). During simulated driving, the MTG is activated (Calhoun et al., 2002). In the present study, we detected power decreases in the MTG and PCiG, which may reflect activation of the MTG and PCiG. We calculated the level of power decrease by comparing passive viewing and active driving. The comparison matched the visual input but differed in terms of motor activity; accordingly, the power decrease may represent visuomotor integration, including higher-order visual processes. Our results showed that the alpha rhythm was prominent in the MTG during driving. Previous studies with driving suggested that visual cognition tasks (Klimesch, 1997; Pfurtscheller and Lopes da Silva, 1999a; Bastiaansen et al., 2001) induced alpha activity in the occipitotemporal area and that the alpha rhythm was associated with higher-order visual processing. Alpha oscillation in the MTG was enhanced after 30 min of passive and active driving. This finding is unexpected and appears to imply that brain activity increases with drowsiness enhancement. We speculate that this process represents a loading-up activity in the MTG to fight off drowsiness. However, we have no direct data to test this speculation, and additional studies are needed. In addition, we speculate that the reason why only alpha oscillation in the MTG is moderately correlated with subjects’ drowsiness and fatigue is that the temporal oscillatory change in MTG is relatively large in comparison with that in other regions, and the MTG is prone to reflect subjective evaluation. The PCiG showed a transient activation between various time points during simulated driving (Calhoun et al., 2002), and this activity was proposed to be involved in switching tasks. Thus, power decrease in the PCiG may reflect a role in the task-switching function during driving.

The sample size was small and included few women. The effect size of MEG was small. Our results require careful generalization, a limitation of the study. The subjects were university students and academics, who were sufficiently educated and intelligent to perform the experimental tasks. However, we did not consider the subjects’ smoking and drinking status, which may influence drowsiness and MEG data. The measurement time in the day varied between subjects. Some subjects underwent the test in the morning and others in the afternoon. There is large circadian variation during the day, which affects drowsiness and MEG readings. Indeed, subjects’ facial expressions already indicated slight drowsiness after 3 min. This circumstance may have led to a discrepancy in drowsiness measures between facial expression recording and MEG recording. According to facial expression recording, half of the subjects dozed off and not all subjects could continue the simulated driving task for 60 min; however, as per the MEG recording, all subjects performed the task for 60 min.

## CONCLUSION

We showed that SFG, IPL, PoCG, MTG, and PCiG were activated during simulated driving. Power increase in the theta band in the SFG may play a role in attention. Power decrease in the right IPL may reflect selectively divided attention and visuospatial processing, whereas that in the left PoCG reflects sensorimotor activation related to driving manipulation. Power decrease in the MTG and PCiG may be associated with object recognition. In the present study, we detected oscillatory differences in specific brain regions that reflected psychological processes involved in driving a vehicle. These oscillatory differences can be useful indices for evaluating drivers’ cognitive preparedness to drive a car, and our findings may help to develop new techniques to prevent car accidents and protect drivers’ lives.

## Conflict of Interest Statement

The Guest Associate Editor Ryouhei Ishii declares that, despite being affiliated to the same institution as authors Kotoe Sakihara, Masayuki Hirata, Seong Yi Ryu, Yoshiyuki Kono, Nozomi Muto, Masako Yoshioka, Toshiki Yoshimine and Shiro Yorifuji, the review process was handled objectively and no conflict of interest exists. The authors declare that the research was conducted in the absence of any commercial or financial relationships that could be construed as a potential conflict of interest.

## Footnotes

1 http://www.fil.ion.ucl.ac.uk/spm/software/spm2/

2 http://www.mathworks.com/

3 http://www.sph.umich.edu/ni-stat/SnPM/

4 https://www.jiscmail.ac.uk/cgi-bin/webadmin?A0=mri3dX
